# Immunoglobulin E Detection Method Based on Cascade Enzymatic Reaction Utilizing Portable Personal Glucose Meter

**DOI:** 10.3390/s21196396

**Published:** 2021-09-24

**Authors:** Hyogu Han, Junhyun Park, Jun Ki Ahn

**Affiliations:** 1 Material & Component Convergence R&D Department, Korea Institute of Industrial Technology (KITECH), Ansan 15588, Korea; ninehyo@kitech.re.kr (H.H.); jhpark@kitech.re.kr (J.P.); 2 Department of Chemistry, Gangneung-Wonju National University, Gangneung 25457, Korea; 3 Department of Biological Engineering, College of Engineering, Konkuk University, Seoul 05029, Korea

**Keywords:** immunoglobulin E, alkaline phosphatase, cascade enzymatic reaction, personal glucose meter, biosensor

## Abstract

We herein describe a cascade enzymatic reaction (CER)-based IgE detection method utilizing a personal glucose meter (PGM), which relies on alkaline phosphatase (ALP) activity that regulates the amount of adenosine triphosphate (ATP). The amount of sandwich assay complex is determined according to the presence or absence of the target IgE. Additionally, the ALP in the sandwich assay catalyzes the dephosphorylation of ATP, a substrate of CER, which results in the changes in glucose level. By employing this principle, IgE was reliably detected at a concentration as low as ca. 29.6 ng/mL with high specificity toward various proteins. Importantly, the limit of detection (LOD) of this portable PGM-based approach was comparable to currently commercialized ELISA kit without expensive and bulky analysis equipment as well as complexed washing step. Finally, the diagnostic capability of this method was also successfully verified by reliably detecting IgE present in a real human serum sample with an excellent recovery ratio within 100 ± 6%.

## 1. Introduction

Food allergy is a deleterious immune response against a component present in food and frequently occurs in many people worldwide [1,2]. Most allergy symptoms cause minor problems; however, sometimes, they can lead to severe illness or physical disability. The sensitization phase of hypersensitivity reactions involved in the biochemical mechanism of food allergy regulates the production of immunoglobulin E (IgE), which is a specific allergen contained in food [3,4,5]. Under normal conditions, IgE level in blood serum is as low as 50–500 ng/mL [6,7,8], whereas immunoglobulin protein level of others, such as IgG, is approximately 10 mg/mL [9]. Its concentration is rapidly elevated upon exposure to allergens and consequently irritates the nose, throat, sinuses, lungs, or ears [10,11]. As the acute anaphylactic reaction caused by IgE is life-threatening [8,12], it is highly desirable to develop a technology to monitor IgE levels in blood serum using simple and cost-effective analytical methods.

Recently, monitoring IgE levels using conventional technologies, such as radioimmunoassay (RIA) [13,14,15], enzyme-linked immunosorbent assay (ELISA) [8,12], and electrochemiluminescence (ECL) immunoassay [16,17,18], have been carried out in many clinical laboratories. RIA is the most widely used diagnostic tool that employs a radiolabeled anti-human antibody for enhanced sensitivity. However, the RIA method has several limitations, including the need for expensive instrumentation, hazardous radioactive materials, and difficulty in quantitative assays, thereby impeding its widespread application in recent years. ELISA is a safer and easier alternative for RIA and is the most widely used clinical diagnostic method for fluorescent or colorimetric detection of various biomarkers. Recently, ECL methods, which are an integration of electrochemistry and chemiluminescent technology, have been extensively used in the clinical field because of their high sensitivity and specificity, low background noise, wide dynamic range, and excellent stability. However, both ELISA and ECL require bulky and expensive equipment, thereby limiting their applications in point-of-care testing (POCT) [19,20].

To overcome these limitations, Xiang et al. developed a novel personal glucose meter (PGM)-based sensing system for detecting various non-glucose targets, such as nucleic acids, proteins, enzymes, and small biological molecules, and has several advantages, including portability, cost-effectiveness, and simplicity [21,22,23,24,25,26,27,28,29,30,31,32]. In this method, an invertase-conjugated DNA probe is used to detect the concentration of glucose generated from sucrose depending on the presence or absence of target molecules in the analyte. However, this approach still has some limitations that restrict its widespread application as a POC biosensor. First, the enzyme conjugation step at the end of the DNA probe should be performed, thereby reducing enzyme activity due to structural hindrance and exclusive utilization owing to individual invertase-modified probes depending on the target molecule.

As an alternative to invertase-based methods, Ahn et al. proposed a label-free and washing-free biosensing method based on a cascade enzymatic reaction (CER) combined with hexokinase and pyruvate kinase [33]. In this system, the target adenosine triphosphate (ATP) serves as a substrate for CER mediated by hexokinase and pyruvate kinase, leading to changes in glucose levels that are measurable by a portable PGM. Recently, CER-based methods have been used in various analyses, including enzyme activity assays and PCR amplification monitoring [34,35,36]. As described above, PGM-based biosensors exhibit infinite potential and versatility in the field of POCT.

In this study, we described a CER-based IgE detection method utilizing PGM, which relies on alkaline phosphatase (ALP) activity to regulate the amount of ATP. This method basically depends on the PGM-based ALP assay system [34], however, this method significantly differs from the previous study [34] in that a target protein (human IgE) is detected utilizing an ALP-modified antibody. The method relies on a sandwich assay that requires the presence of target IgE, including an ALP-modified secondary antibody, which decomposes ATP, and the substrate for the CER reaction, thereby inducing changes in glucose concentration. In addition, magnetic separation steps are employed in this method instead of the conventional ELISA plate washing step. Finally, we successfully detected the target IgE by simply reading the glucose signal using portable PGM, without expensive and bulky analysis equipment, even in a facility-limited environment through employing the proposed strategy that could overcome the challenges in conventional fluorescent or colorimetric ELISA.

## 2. Materials and Methods

### 2.1. Materials

D-Glucose, magnesium chloride (MgCl_2_), diethanolamine (DEA), 2-(N-morpholino)ethanesulfonic acid monohydrate (MES), tris (hydroxymethyl) aminomethane hydrochloride (Tris-HCl), phosphoenolpyruvic acid (PEP), adenosine 5′-triphosphate disodium salt hydrate (ATP), casein, sodium chloride (NaCl), hexokinase, pyruvate kinase, Tween-20, bovine serum albumin (BSA), human serum albumin (HSA), human serum, *p*-nitrophenyl phosphate (*p*-NPP) substrate solution, 3,3′,5,5′-tetramethylbenzidine (TMB) substrate solution, rabbit anti-goat IgG antibody-alkaline phosphatase (Ab-ALP) were purchased from Sigma-Aldrich (St. Louis, MO, USA). Target human IgE was supplied by NIBSC (Hertfordshire, UK). Mouse anti-human IgE antibody-alkaline phosphatase (Ab-ALP) was purchased from SouthernBiotech (Birmingham, AL, USA). Goat anti-human IgE antibody purchased form Thermo-Fisher Scientific (Waltham, MA, USA). Troponin I cardiac antibody (anti-cTnI) was purchased from Hytest (Turku, Finland). Human IgE ELISA core kit was purchased from Komabiotech (Seoul, Korea). Aqueous solutions were prepared by use of ultrapure DNase/RNase-free distilled water (DW) purchased from Bioneer (Daejeon, Korea). Carboxyl-modified magnetic nanoparticles (MNPs, diameter of 300 nm) were provided by Amo Lifescience (Gimpo, Korea). The portable glucose meter (PGM) was purchased from Accu-Chek (Roche, Basel, Switzerland).

### 2.2. Preparation of Capture Particle

First, 200 μL of MNPs (10 mg/mL) was added to a 1.5 mL microtube, the supernatant was discarded using a magnetic separator, and resuspended in 133.4 μL MES buffer (50 mM MES, pH 6.0). Then, 66.6 μL goat anti-human IgE antibody (primary antibody) (1.5 mg/mL) and 100 μL EDC (1 mg/mL in MES buffer) were added and incubated at room temperature for 30 min. After the reaction, the mixture was washed three times with 1 mL of PBS-T (1X PBS (137 mM NaCl, 2.7 mM KCl, 10 mM Na_2_HPO_4_, 1.8 mM KH_2_PO_4_) with 0.1% Tween-20, pH 7.4) and resuspended and incubated at 4 °C for 12 h for stabilization. The stabilized capture particles (CPs) were stored at 4 °C after being suspended in TBS-T (20 mM Tris-HCl, 154 mM NaCl, 0.05% Tween-20, pH 7.4) in a concentration of 2 mg/mL.

### 2.3. Characterization of Capture Particle

The antibody immobilization statuses of the CPs were analyzed using anti-goat IgG antibody-alkaline phosphatase. First, 10 μL CPs (2 mg/mL), 30 μL rabbit anti-goat IgG antibody-alkaline phosphatase (2 μg/mL) or mouse anti-human IgE antibody-alkaline phosphatase (secondary antibody) (2 μg/mL) and 160 μL tris buffer (50 mM Tris-HCl, 1 mM MgCl_2_, pH 7.4) were added to a 1.5 mL microtube and incubated at room temperature for 30 min. The CPs were washed three times with 1 mL of PBS-T and then transferred to microplate well. After the addition of 100 μL of *p*-NPP substrate solution to the well, the absorbance was measured at 405 nm using the Infinite M 200 PRO (Tecan, Switzerland) after 10 min. The MNPs and CPs were analyzed to verify the modification of the antibody on the surface using Perkin Elmer Spectrum BX FT-IR System (Perkin Elmer, Waltham, MA, USA).

### 2.4. Formation of a Magnetic Bead-Based Sandwich Complex

Target human IgE (50 μL) at various concentrations or human IgG, BSA, HSA, anti-cTnI (10 μg/mL) was first mixed with 50 μL of Mouse anti-human IgE Fc-alkaline phosphatase (2 μg/mL) and incubated at room temperature for 30 min; then, 50 μL of CPs (2 mg/mL) was added and incubated for another 30 min. The CPs were collected with a magnet and washed four times with 100 μL of TBS-T.

### 2.5. Human IgE Assay Based on CER Utilizing PGM

After discarding the supernatant of the sandwich complexes prepared in 2.4., the CPs were resuspended in 40 μL of reaction mixture I (20 mM Tris-HCl, 0.16 mM MgCl_2_, 60 mM DEA, 1 mM ATP, pH 9.8) and incubated at 37 °C for 2 h. Next, 30 μL of the above reaction solution was transferred to a PCR tube, 20 μL of reaction mixture II (250 mM Tris-HCl, 25 mM MgCl_2_, 12.5 mM D-glucose, 5 mM PEP, 1 unit pyruvate kinase, 1 unit hexokinase, pH 7.4) was added and then incubated at 30 °C for 30 min. Finally, the glucose level of the reactant was measured by a PGM.

### 2.6. Human IgE Assay Based on Colorimetric p-NPP

Transferred 100 μL of the sandwich complexes prepared in Section 2.4 to the microplate well. After the addition of 100 μL of *p*-NPP substrate solution to the well, the absorbance was measured at 405 nm using the Infinite M 200 PRO after 1 h.

### 2.7. Commercialized ELISA Kit

Primary antibody (100 μL) (included in kit) (2 μg/mL) was added to each well and incubated at 4 °C for overnight. After the antibody was coated, it was washed three times using a 300 μL of PBS-T and then washed in the same manner at the end of each step. To prevent non-specific binding of well surfaces, 200 μL of blocking solution (1X PBS with 0.1% casein) was added to each well and incubated at room temperature for 1h. After washing step, 100 μL of human IgE at various concentrations was added, incubated at room temperature for 1 h, and the washing step was repeated. Then, 100 μL of horseradish peroxidase (HRP)-modified secondary antibody (200 ng/mL) was added, and the mixture was incubated at room temperature for 1 h. After the washing step, 100 μL of TMB substrate solution was added to the wells and then incubated at room temperature for 20 min. Finally, to terminate the reaction, 100 μL of stop solution (2M H_2_SO_4_) was added, the absorbance was measured at 450 nm using the Infinite M 200 PRO.

### 2.8. Zeta Potential Analysis of Capture Particles and Proteins

The zeta potential of CPs and other proteins of used in Section 2.4 was measured with a Litesizer 500 (Anton Paar, Graz, Austria) at room temperature after dilution and suspension in tris buffer to measurement. The concentration of CPs, human IgE, human IgG, BSA, HSA, and Ab-ALP were 0.4, 1.6, 1.0, 0.8, 1.2, and 0.01 mg/mL, respectively.

### 2.9. Recovery Test

Real sample-mimicking samples were prepared by spiking human IgE at varying concentrations into non-diluted human serum (containing 1 mg/mL human IgG) and analyzed directly following the same detection procedure used in the buffer solution. The calibration curve was created with a set of standards where human IgE at a known amount is contained in the non-diluted human serum. Based on the calibration curve, the unknown amount of human IgE was examined.

## 3. Results and Discussion

### 3.1. Detection Principle of the PGM-Based Human IgE Assay

**Figure 1:**
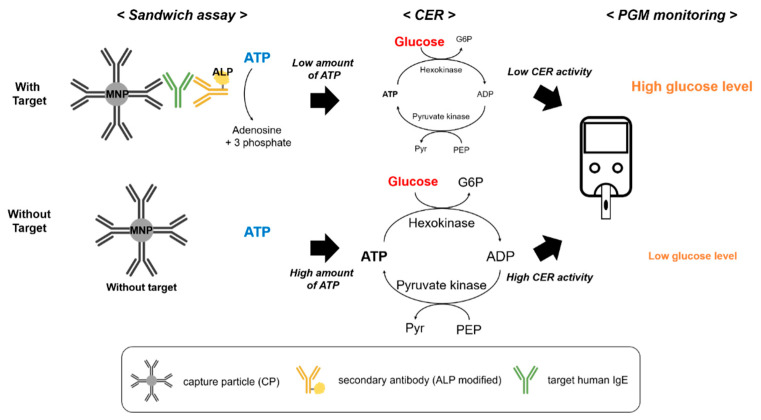
Schematic illustration of the IgE detection method based on a CER utilizing portable personal glucose meter. The overall assay process involves the formation of a sandwich assay comprising CP, target IgE, and ALP-modified secondary antibody, as well as CER promoted by residual ATP, which is decomposed by ALP.

The conceptual design of the PGM-based human IgE assay is shown in Figure 1. This strategy to detect human IgE with PGM is based on a previously reported cascade enzymatic reaction [33], where the combination of hexokinase and pyruvate kinase promotes multiple rounds of ATP generation and consumption, leading to highly reduced glucose levels. In the absence of target IgE, the ALP-modified secondary antibody is eliminated by a magnetic-based washing step, resulting in a lack of influence of the initial ATP concentration that allows hexokinase to promote the conversion of glucose to glucose-6-phosphate and ATP to ADP by offering a phosphate group from ATP to glucose. The consequently produced ADP enables pyruvate kinase to catalyze the conversion of both phosphoenolpyruvic acid (PEP) to pyruvate and ADP to ATP by providing the phosphate group from PEP to ADP. The reproduced ATP is again utilized as a substrate for multiple rounds of CERs, leading to a highly reduced glucose concentration. On the other hand, the presence of target IgE allows the formation of a sandwich assay comprising CP, target IgE, and ALP-modified secondary antibody; further, the sandwich complexes are separated using a magnet. Then, the ALP of the secondary antibody cleaves the phosphate of ATP, leading to the suppression of subsequent CER. As a result, the glucose level remained high with a smaller reduction than when the target IgE was not present, and the amount of glucose, proportional to the target IgE, was simply detected by portable PGM.

### 3.2. Characterization of Capture Particle

**Figure 2:**
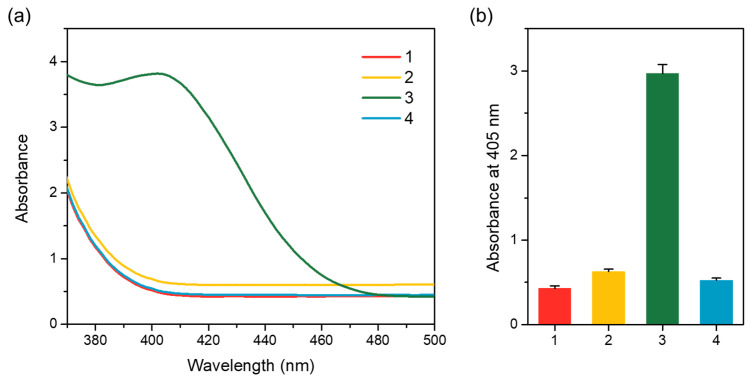
Characterization of antibody immobilization of CPs based on colorimetric method utilizing *p*-NPP substrate. Absorbance spectra (**a**) and absorbance intensity at 405 nm (**b**) of (1) bare magnetic particles (MNPs), (2) CPs, (3) CPs with anti-goat IgG Ab-ALP, and (4) CPs with secondary antibody (mouse anti-human IgE Ab-ALP).

CPs were prepared by chemical conjugation of primary antibody with MNPs. As shown in Figure 2, low absorbance intensities were observed for the bare MNPs and CPs. On the contrary, a significantly high absorbance intensity at 405 nm was observed in the CPs that reacted with ALP-modified anti-goat IgG antibody. It seems that the ALP-modified anti-goat IgG antibody is specifically bound to the primary antibody, and a yellow color was produced due to the dephosphorylation of *p*-NPP by ALP. In addition, the absorbance of CPs reacted with secondary antibody was similar to the absorbance of bare MNPs or CPs, indicating that a non-specific reaction does not occur between the prepared CPs and secondary antibody. Moreover, antibody conjugation to MNPs was confirmed by FTIR analysis. As shown in Figure S1, the peak around 1637 cm^−1^ reflected the C = O bond and antibody amide I, and the peak at 1550 cm^−1^ was assigned to the N–H bond of antibody amide II. These results confirmed that the antibodies were covalently bound to the MNPs surface.

### 3.3. Detection Feasibility of the PGM-Based Human IgE Assay

**Figure 3:**
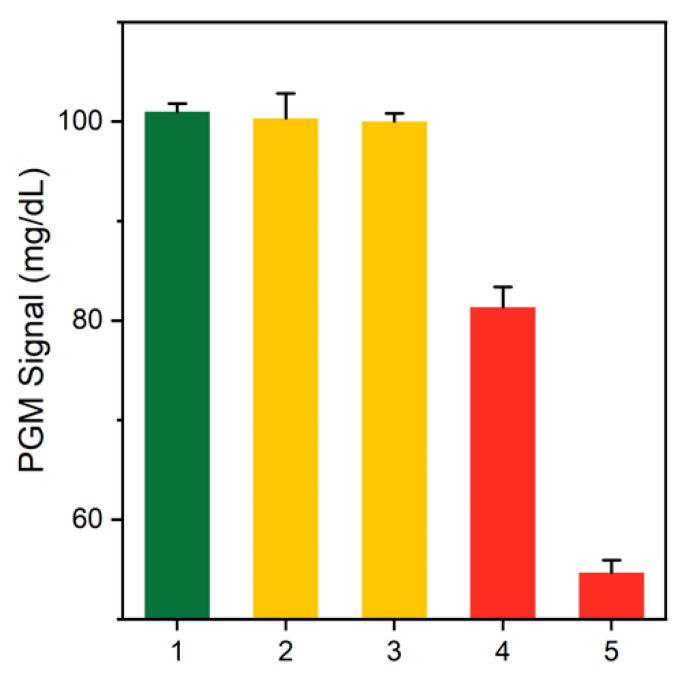
Detection feasibility of the PGM-based IgE assay. The PGM signals from different samples containing (1) no enzyme with target IgE, (2) only hexokinase with target IgE, (3) only hexokinase without target IgE, (4) both hexokinase and pyruvate kinase with target IgE, and (5) both hexokinase and pyruvate kinase without target IgE. The concentrations of target IgE, hexokinase, and pyruvate kinase were 1 μg/mL, 50 unit/mL, and 50 unit/mL, respectively.

The detection feasibility of the PGM-based human IgE assay was validated by measuring glucose levels using PGM in different samples. As shown in Figure 3, significantly decreased glucose levels were observed in the absence of target IgE, because intact ATP activated CER. However, in the presence of target human IgE, ATP was reduced by ALP, and CER was subsequently inhibited, thereby resulting in a higher glucose level, compared to the intact amount of ATP. In addition, when glucose was reduced only by hexo-kinase and not CER, there was no significant difference, according to the presence or absence of target human IgE. These results confirm that CER promoted by hexokinase and pyruvate kinase induces a substantial decrease in glucose levels.

To optimize the detection performance of the PGM-based IgE assay, the enzyme concentrations (hexokinase and pyruvate kinase) and the CER time were investigated. First, we optimized the concentrations of hexokinase and pyruvate kinase. Enzymes at too low a concentration may inhibit the CER. Conversely, those at too high a concentration may provide the small signal difference between the presence and absence of a target IgE, which causes low sensing capability. As shown in Figure S2, enzymes over 2.5 units allowed the PGM signal to saturate within just 5 min, but enzymes lower than 0.5 units produced CER activity that was too low. Next, the optimal CER time was examined by measuring the change in PGM signals before and after CER in the presence of the target IgE.

The results demonstrated that the change in the PGM signal increased with increasing reaction time up to 20 min but decreased over 20 min (Figure S3). Based on the results, 1 unit for each enzyme and 20 min CER time were chosen for further experiments.

### 3.4. Sensitivity of the PGM-Based Human IgE Assay

**Figure 4:**
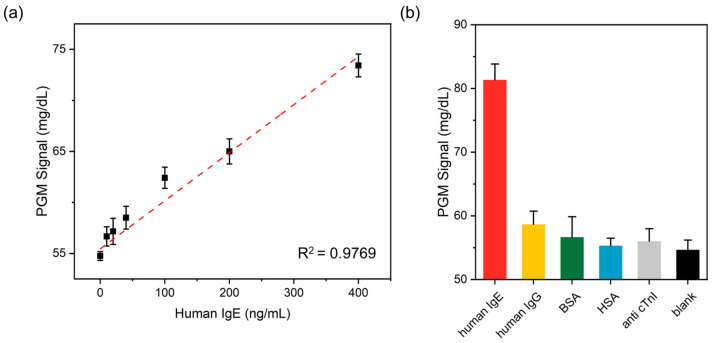
(**a**) The linear relationship between the PGM signal and target IgE concentration within the range of 0 to 400 ng/mL. (**b**) Detection selectivity of the IgE detection system. The concentrations of IgE and other antibodies (IgG, BSA, HSA, and anti-cTnI) were 1 μg/mL and 10 μg/mL, respectively. The *p*-values (unpaired two-tailed *t*-test) of IgG, BSA, HSA, and anti-cTnI toward IgE were 0.0003, 0.0005, 0.0001, 0.0002, and 0.0001, respectively. *p*-values lower than 0.05 were considered significant.

The sensitivity of the PGM-based human IgE assay was determined by measuring glucose level as a function of human IgE concentration. As shown in Figure 4a, the glucose level increased with the increasing concentration of human IgE. A strong linear relationship (R^2^ = 0.9796) existed in the range of 10–400 ng/mL, and the limit of detection (LOD) (3σ/slope) was ca. 29.59 ng/mL, which is comparable to or higher than the limits of other human IgE assay methods (Table 1) [37,38,39,40,41,42,43,44,45,46,47]. Notably, the detection sensitivity of our strategy was sufficient to detect clinically important human IgE concentrations in human serum (>240 ng/mL) [48].

Furthermore, we compared the sensitivity of our method to the colorimetric *p*-NPP-based method and the commercialized ELISA kit. By reacting the sandwich complex formed by the CP and the secondary antibody with the *p*-NPP substrate solution, a color change of the *p*-NPP substrate solution to yellow was observed in proportion to the target IgE concentration (Figure S4a), and the LOD of this method was estimated at ca. 0.52 ng/mL (Figure S5). In addition, we detected IgE by utilizing a commercialized ELISA kit based on HRP and TMB substrate solution, where LOD was measured at ca. 0.26 ng/mL (Figures S4b and S6). Although the PGM-based IgE assay showed lower sensitivity than both the *p*-NPP-based method and the ELISA kit, this method could be simply operated by a hand-held PGM without the requirement of expensive and bulky analysis equipment, such as a microplate reader.

### 3.5. Selectivity of the PGM-Based Human IgE Assay

The selectivity of the present method for the PGM-based human IgE assay was also investigated by the addition of human IgE and four interfering proteins, including human IgE, BSA, HSA, and anti-cTnI. As shown in Figure 4b, a high PGM signal was only observed in the sample containing human IgE. On the other hand, the presence of interfering proteins was similar to that in the absence of human IgE, even though their concentrations were ten times higher than that of human IgE (*p* < 0.05; unpaired two-tailed *t*-test). In addition, the zeta potential of CPs and interfering proteins was investigated. As shown in Table S1, both CPs and interfering proteins have weak negative charges, so the interference by electrostatic attraction is also considered negligible. These results clearly demonstrated satisfactory selectivity of our human IgE detection method.

### 3.6. Practical Applicability of the PGM-Based Human IgE Assay

The human IgE contained in non-diluted human serum was analyzed to demonstrate the practical applicability of the PGM-based human IgE detection method. Figure S7 showed that human IgE in non-diluted serum was examined based on the standard addition method [49,50]. As a result, glucose levels measured by PGM increased as the concentration of IgE in serum increased from 0.05 to 5 μg/mL. In addition, a coefficient of variation (CV) of less than 8% and a recovery ratio between 99% and 105% were observed, verifying the high reproducibility and precision of this method (Table 2). Based on these results, it was demonstrated that the IgE detection system developed in this study could be used for reliable detection of the IgE concentration in human serum.

## 4. Conclusions

In the study described above, we proposed an IgE detection method-based portable PGM that serves as an analysis equipment to quantitatively detect target IgE. Our strategy employs CER combined by hexokinase and pyruvate kinase, leading to changes in glucose level that is measurable by a portable PGM. Our PGM-based method to detect IgE can be conducted even in facility-limited environment because it does not need a bulky and expensive device that is generally needed in conventional fluorescent and colorimetric methods, which enables the realization of convenient and cost-effective system to detect IgE. By employing this design principle, IgE was detected quantitatively with high comparable sensitivity to recent reports and commercialized ELISA assay kit. The practical utility of the proposed methods was successfully verified by reliably determining IgE present in human serum samples. The basic signaling strategy developed in this study would serve as a new platform to construct novel immunoassay systems to detect other various biological molecules.

## Figures and Tables

**Table 1:** Summary of the previously reported IgE detection methods.

Method	Key Elements	LOD (ng/mL)	Sample	Ref.
Arrays	Indirect assay with bound allergens	49.3	Non-diluted serum	[37]
Electrochemical	Aptasensor	300	Non-diluted serum	[38]
Immunochemical	Paper-based assay	2.4	Non-diluted serum	[39]
	Vertical flow assays	1900	Diluted serum (10%)	[40]
Label-free	SPR	190	Buffer solution	[41]
Microfluidics	Miniaturized array	27	Non-diluted serum	[42]
		2.4	Non-diluted serum	[43]
	Miniaturized immunodiffusion	1	Diluted serum (20%)	[43]
Nanomaterial-based	Magnetic capture	24	Diluted serum	[44]
	Quantum dots	84	Diluted serum (2%)	[45]
	Silver particle	20	Diluted serum (20%)	[46]
PGM	Cascade enzymatic reaction	29.6	Non-diluted serum	This work

**Table 2:** Determination of human IgE spiked into non-diluted human serum. Based on the calibration curve (Figure S7), the concentration of human IgE in non-diluted human serum was determined using the PGM signals from unknown samples. SD, CV, and recovery are defined as standard deviation of three measurements, coefficient of variation (SD/mean × 100), and measured value/added value × 100, respectively.

Added IgE (μg/mL)	Measured IgE (μg/mL)	SD	CV (%)	Recovery (%)
1.0	1.05	0.082	7.75	105.21
0.5	0.49	0.036	7.35	99.48

## Supplementary Materials

The following are available online at https://www.mdpi.com/article/10.3390/s21196396/s1. Figure S1: FTIR spectra of MNPs and CPs. Figure S2: The PGM signals obtained from varying concentration of each hexokinase and pyruvate kinase in the absence of IgE. Figure S3: The PGM signal change at different CER reaction times in the presence of IgE. Figure S4: (a) Colorimetric IgE ELISA method scheme based on the dephosphorylation of *p*-NPP promoted by ALP. (b) Commercialized IgE ELISA kit principle based on the oxidation of TMB promoted by HRP. Figure S5: The sensitivity of the IgE assay system based on dephosphorylation of *p*-NPP promoted by ALP modified with secondary IgE. Figure S6: Sensitivity of IgE assay kit based on TMB oxidation promoted by HRP modified with secondary IgE. Figure S7: The PGM signal as a function of target IgE spiked in non-diluted human serum. Table S1: Zeta potential of different samples.
