# Supplementary material for: Immunoglobulin E Detection Method Based on Cascade Enzymatic Reaction Utilizing Portable Personal Glucose Meter
Source: Sensors (Basel). 2021 Sep 24;21(19):6396. doi: 10.3390/s21196396 (PMC8513091; doi:10.3390/s21196396)
Supplement: Supplementary file 1 [file sensors-21-06396-s001.zip › sensors-1365474-SI.pdf]

sensors

Article

Immunoglobulin E Detection Method Based on Cascade Enzymatic Reaction Utilizing Portable Personal Glucose Meter

Hyogu Han [1,2], Junhyun Park [1,3] and Jun Ki Ahn [1,*]

1 Material & Component Convergence R&D Department, Korea Institute of Industrial Technology (KITECH), Ansan 15588, Korea; ninehyo@kitech.re.kr (H.H.); jhpark@kitech.re.kr (J.P.)
2 Department of Chemistry, Gangneung-Wonju National University, Gangneung 25457, Korea
3 Department of Biological Engineering, College of Engineering, Konkuk University, Seoul 05029, Korea
* Correspondence: jkahn@kitech.re.kr; Tel.: +82-31-8040-6229

1. Supplementary Materials

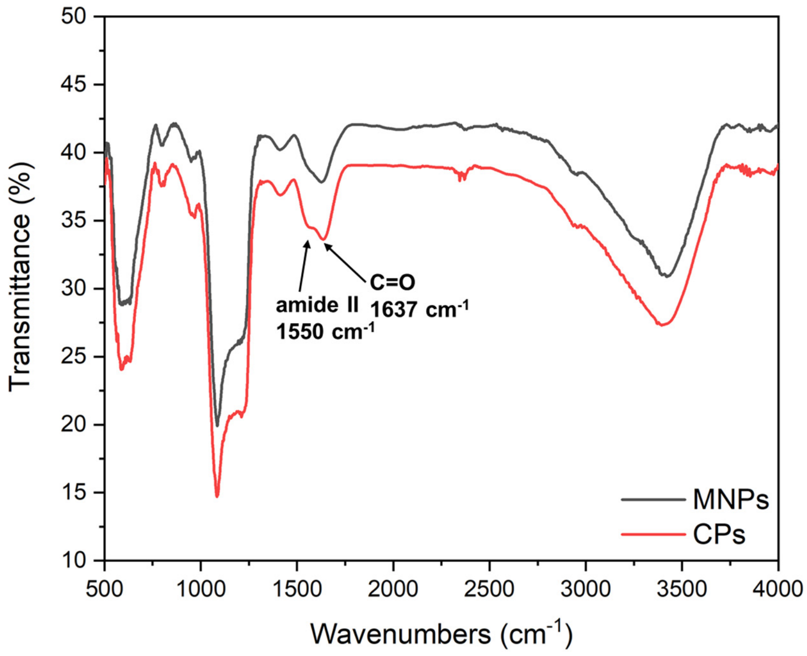

Figure S1. FTIR spectra of MNPs and CPs.

Citation: Han, H.; Park, J.; Ahn, J.K. Immunoglobulin E Detection Method Based on Cascade Enzymatic Reaction Utilizing Portable Personal Glucose Meter. *Sensors* **2021**, *21*, 6396. https://doi.org/10.3390/s21196396

Academic Editor: James F. Rusling.

Received: 18 August 2021
Accepted: 23 September 2021
Published: 24 September 2021

Publisher's Note: MDPI stays neutral with regard to jurisdictional claims in published maps and institutional affiliations.

Copyright: © 2021 by the authors. Licensee MDPI, Basel, Switzerland. This article is an open access article distributed under the terms and conditions of the Creative Commons Attribution (CC BY) license (http://creativecommons.org/licenses/by/4.0/).

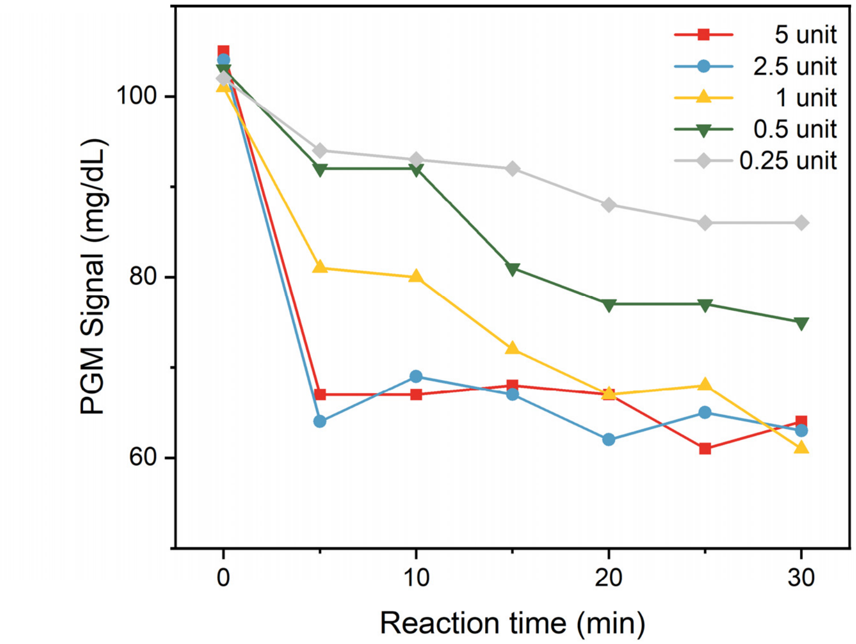

Figure S2. The PGM signals obtained from varying concentration (0.025, 0.5, 1, 2.5, and 5 units) of each hexokinase and pyruvate kinase in the absence of IgE.

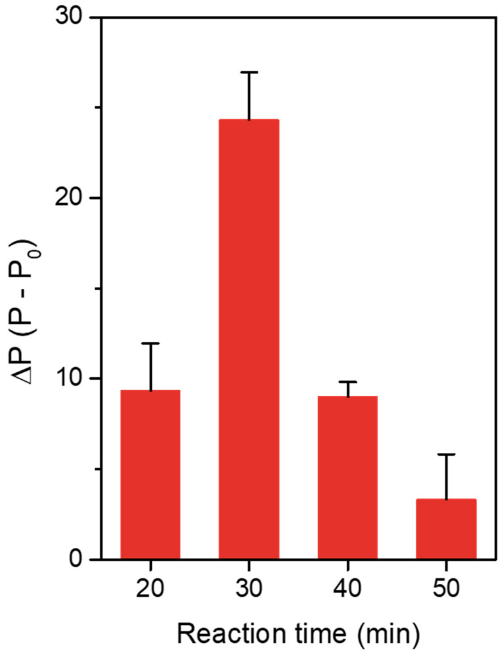

Figure S3. The PGM signal change (ΔP) at different CER reaction times (20, 30, 40, and 50 min) in the presence of IgE (1 μg/mL). ΔP is defined as P-P_0, where P and P_0 indicate the PGM signal after and before the CER reaction, respectively. The concentrations of hexokinase and pyruvate kinase were 1 unit.

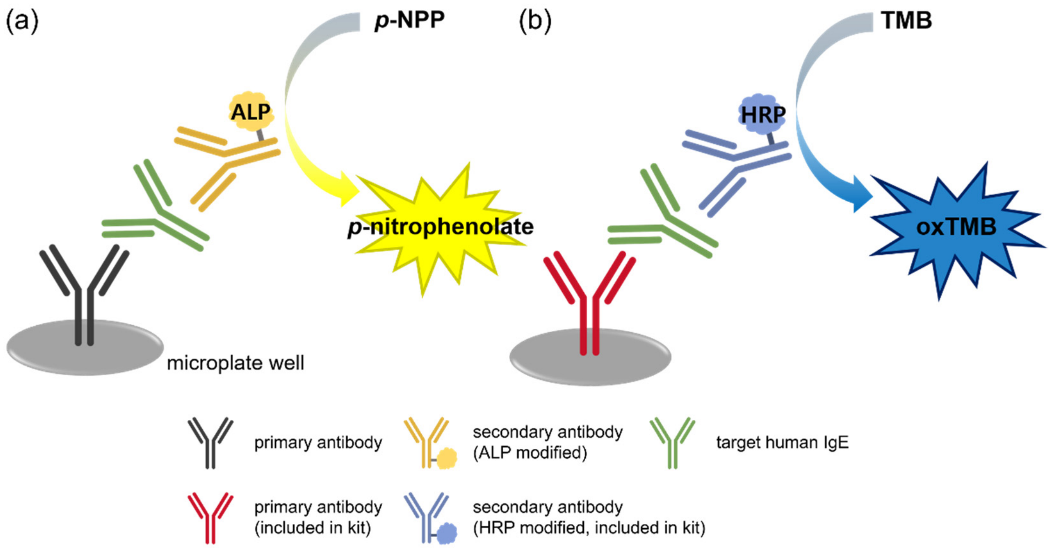

Figure S4. (a) Colorimetric IgE ELISA method scheme based on the dephosphorylation of p-NPP promoted by ALP. (b) Commercialized IgE ELISA kit principle based on the oxidation of TMB promoted by HRP.

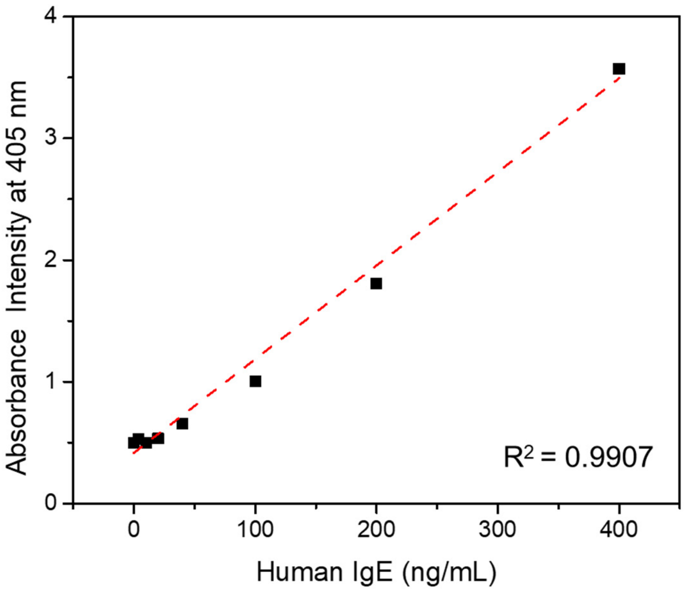

Figure S5. The sensitivity of the IgE assay system based on dephosphorylation of *p*-NPP promoted by ALP modified with secondary IgE. The linear relationship between absorbance intensity at 405 nm and target IgE concentration within the range of 0 to 400 ng/mL.

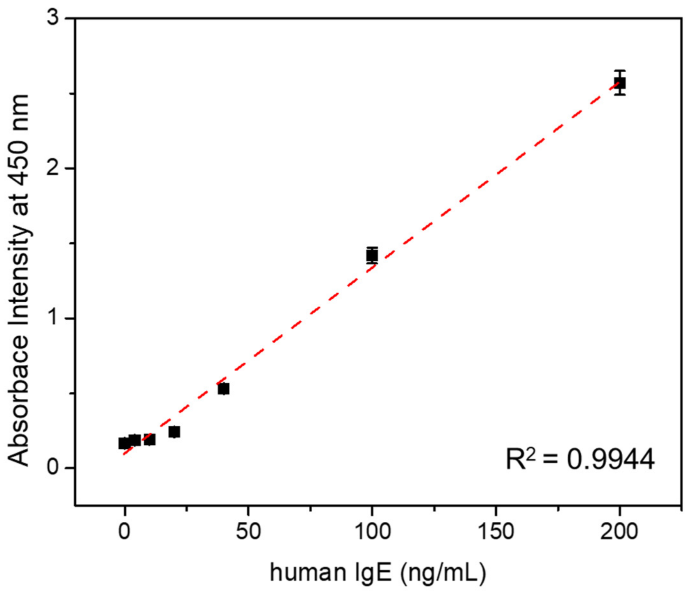

Figure S6. Sensitivity of IgE assay kit (Koma Biotech, Korea) based on TMB oxidation promoted by HRP modified with secondary IgE. The linear relationship between absorbance intensity at 450 nm and target IgE concentration within the range of 0 to 200 ng/mL.

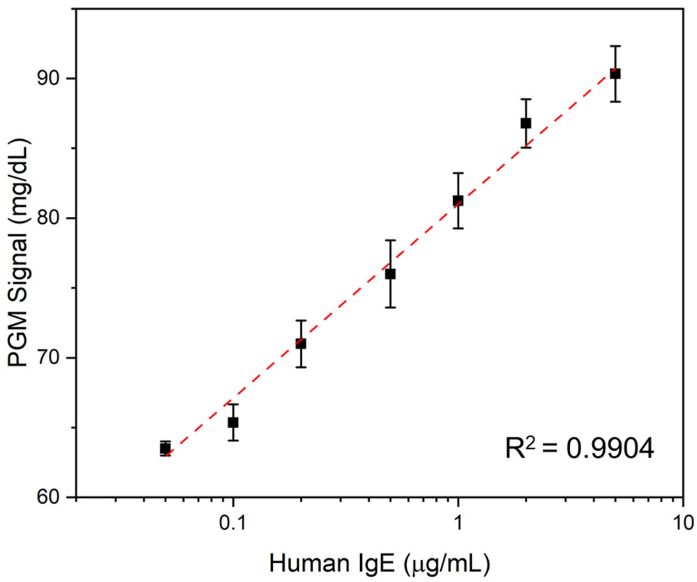

Figure S7. The PGM signal as a function of target IgE spiked in non-diluted human serum. The logarithmic relationship between the PGM signal and target IgE concentration with the range of 0.05 to 5 μg/mL.

Table S1. Zeta potential of different samples.

Sample Name	Zeta Potential (mV)
CPs	-7.30 ± 0.4
human IgE	-8.30 ± 0.36
human IgG	-2.03 ± 0.20
BSA	-2.80 ± 1.66
HSA	-8.57 ± 2.21
Ab-ALP	-5.17 ± 1.18
